# Prenatal Exposure to Ambient Air Pollution and Cerebral Palsy

**DOI:** 10.1001/jamanetworkopen.2024.20717

**Published:** 2024-07-09

**Authors:** Yu Zhang, Yuhong Hu, Robert Talarico, Xinye Qiu, Joel Schwartz, Deshayne B. Fell, Maryam Oskoui, Eric Lavigne, Carmen Messerlian

**Affiliations:** 1 Department of Environmental Health, Harvard T.H. Chan School of Public Heath, Boston, Massachusetts; 2 Environmental Health Science and Research Bureau, Health Canada, Ottawa, Ontario, Canada; 3 School of Epidemiology and Public Health, University of Ottawa, Ontario, Canada; 4 Department of Epidemiology, Harvard T. H. Chan School of Public Health, Boston, Massachusetts; 5 Children’s Hospital of Eastern Ontario Research Institute, Ottawa, Ontario, Canada; 6 Department of Pediatrics, McGill University, Montreal, Quebec, Canada; 7 Department of Neurology and Neurosurgery, McGill University, Montreal, Quebec, Canada; 8 ICES, Ottawa, Ontario, Canada; 9 Massachusetts General Hospital Fertility Center, Department of Obstetrics and Gynecology, Boston; 10 Now with Pfizer, Kirkland, Quebec, Canada

## Abstract

**Importance:**

Air pollution is associated with structural brain changes, disruption of neurogenesis, and neurodevelopmental disorders. The association between prenatal exposure to ambient air pollution and risk of cerebral palsy (CP), which is the most common motor disability in childhood, has not been thoroughly investigated.

**Objective:**

To evaluate the associations between prenatal residential exposure to ambient air pollution and risk of CP among children born at term gestation in a population cohort in Ontario, Canada.

**Design, Setting, and Participants:**

Population-based cohort study in Ontario, Canada using linked, province-wide health administrative databases. Participants were singleton full term births (≥37 gestational weeks) born in Ontario hospitals between April 1, 2002, and March 31, 2017. Data were analyzed from January to December 2022.

**Exposures:**

Weekly average concentrations of ambient fine particulate matter with a diameter 2.5 μm (PM_2.5_) or smaller, nitrogen dioxide (NO_2_), and ozone (O_3_) during pregnancy assigned by maternal residence reported at delivery from satellite-based estimates and ground-level monitoring data.

**Main outcome and measures:**

CP cases were ascertained by a single inpatient hospitalization diagnosis or at least 2 outpatient diagnoses for children from birth to age 18 years.

**Results:**

The present study included 1 587 935 mother-child pairs who reached term gestation, among whom 3170 (0.2%) children were diagnosed with CP. The study population had a mean (SD) maternal age of 30.1 (5.6) years and 811 745 infants (51.1%) were male. A per IQR increase (2.7 μg/m^3^) in prenatal ambient PM_2.5_ concentration was associated with a cumulative hazard ratio (CHR) of 1.12 (95% CI, 1.03-1.21) for CP. The CHR in male infants (1.14; 95% CI, 1.02-1.26) was higher compared with the CHR in female infants (1.08; 95% CI, 0.96-1.22). No specific window of susceptibility was found for prenatal PM_2.5_ exposure and CP in the study population. No associations or windows of susceptibility were found for prenatal NO_2_ or O_3_ exposure and CP risk.

**Conclusions and relevance:**

In this large cohort study of singleton full term births in Canada, prenatal ambient PM_2.5_ exposure was associated with an increased risk of CP in offspring. Further studies are needed to explore this association and its potential biological pathways, which could advance the identification of environmental risk factors of CP in early life.

## Key Points

**Question:**

Is ambient air pollutant exposure during pregnancy associated with cerebral palsy (CP) risk among full term births?

**Findings:**

In this cohort study of over 1 million singleton full term births from all hospitals in Ontario, Canada (2002-2017), a per IQR increase in prenatal residential ambient fine particulate matter with a diameter 2.5 μm (PM_2.5_) concentration (2.7 μg/m^3^) was associated with a 1.12 times higher risk of CP.

**Meaning:**

These findings suggest that prenatal interventions to reduce ambient PM_2.5_ exposure are needed to mitigate the potential risk of CP during fetus development.

## Teaser

This cohort study evaluates the association between prenatal exposure to ambient air pollution and risk of cerebral palsy in a cohort of children residing in Canada and born at term gestation.

## Introduction

Cerebral palsy (CP) is the most common cause of physical disability in childhood, representing a group of nonprogressive clinically heterogeneous neurodevelopmental disorders that are characterized by motor impairment. CP appears early in life and leads to lifelong motor disability.^1,2^ The motor disorders of CP are often accompanied by disturbances of sensation, perception, cognition, communication, and behavior, as well as by epilepsy and secondary musculoskeletal problems.^3^ Despite decreases in perinatal and neonatal morbidity and major changes in prenatal and neonatal care, the overall prevalence of CP has remained stable over time at between 1 and 4 per 1000 live births.^4,5,6^ The prevalence, severity, and burden of CP and its comorbidities make prevention a public health priority. The cause of CP, however, has proved complex given its heterogeneity with respect to clinical subtypes and ranges of comorbidities and functional outcomes.^7^ Although preterm birth is one of the known important risk factors for CP, three-fourths of CP cases are born at term and the mechanism of CP among full term births remains elusive.^8^ One recent study in California reported conceptions in winter and spring seasons had higher CP risk than those conceived in summer and fall, and preterm birth only mediated a small proportion of the association, suggesting an environmental cause for CP among full term births.^9^ Recognizing that prenatal and perinatal exposures are greatly linked to the risk of CP among full term births, identifying and understanding potential risk factors provides opportunities for prevention.^7^

Prenatal exposure to air pollution is associated with decelerated neurological development early in life and increased risk of neurodevelopmental problems.^10^ Although no animal or human study has ever reported a direct link between air pollution and CP, it is possible that air pollution may increase the risk of CP following similar pathophysiological pathways.

In animal models, in utero exposure to fine particles is associated with structural changes in white matter, delay in cognitive development, and disruption of neurogenesis.^11,12,13^ Proposed mechanisms for the association between prenatal air pollution and adverse health effects include epigenetic changes,^14^ proinflammatory pathways,^15^ and oxidative stress.^16,17,18,19^ Evidence from human studies is also mounting to support air pollution–related neurodevelopmental disorders (eg, elevated risks of autism and structural brain alterations among other disorders).^20^

Recent evidence showed the transfer of black carbon particles across to the fetal side of the human placenta,^21^ indicating air pollutants in maternal circulation can cross the placenta and directly affect the fetus.^22^ Furthermore, brain development starts as early as the third week of gestation and continues until birth.^23^ Environmental exposures at different gestational weeks can have different effects due to sensitive periods of brain development, which few studies have investigated.

In this study, we leveraged the population-based health administrative data from the province of Ontario, Canada (2002-2017) and assembled a population cohort to examine the associations between prenatal exposure to ambient air pollutants and CP among full term births. We further assessed windows of susceptibility by gestational weeks and heterogeneity by infant sex.

## Method

### Study Population and Data Sources

Ethical approval for this cohort study was obtained from the research ethics boards of Health Canada and the Ottawa Health Science Network. Informed consent was waived because ICES is a prescribed entity under Ontario’s Personal Health Information Protection Act. The study followed the Strengthening the Reporting of Observational Studies in Epidemiology (STROBE) reporting guideline for cohort studies.

We included all full term births (37 or more gestational weeks completed [259 days]) born in hospitals in Ontario, Canada between April 1, 2002, and March 31, 2017, leveraging the administrative health data housed at ICES, an independent, nonprofit research institute, which collects and analyzes health care and demographic data of all legal residents with a valid health card in Ontario, Canada (>99% of the population).^24^ We excluded children from a multiple birth pregnancy, invalid health card numbers, missing 6-digit postal codes for exposure assessment, missing or invalid covariates and exposure estimates, and mothers or children who were ineligible for provincial health insurance (Ontario Health Insurance Plan [OHIP]) at birth (eFigure 1 in Supplement 1). eTable 1 in Supplement 1 summarizes the distribution of gestational weeks at birth among CP cases in all births born in hospitals in Ontario, Canada between 2002 and 2017.

We compiled databases from various sources in our analyses. The Discharge Abstract Database from the Canadian Institute for Health Information (CIHI-DAD), which contains information from all hospital admissions, and the OHIP Records Database, which contains health services billing data from all physicians in Ontario, were used for outcome ascertainment. Demographic information and maternal data were obtained from the Registered Persons Database, which included demographic information on those registered for health insurance, and MOMBABY, an ICES-derived cohort that links hospital admission records of mothers with their newborns for all hospital births in the province based on records from CIHI-DAD. Area-level socioeconomic status variables were obtained from the Ontario Marginalization Index (ON-Marg), which is a specific version of the Canadian Marginalization Index that explores multiple dimensions of social and economic marginalization in Ontario.^25^

### Exposure Assessment

Prenatal residential exposures to ambient air pollutants, including fine particulate matter with a diameter 2.5 μm or less (PM_2.5_), nitrogen dioxide (NO_2_), and ozone (O_3_), were assigned to the study population as the estimated weekly concentrations at the geographic center of each 6-digit postal code area, using the reported residential address at delivery. Weekly average concentrations of residential air pollutants during pregnancy were obtained using the recorded gestational age available in the MOMBABY dataset. Details on the measurements of ambient air pollutants were described in a previously published study.^24^

In brief, annual PM_2.5_ concentrations were estimated incorporating data from geophysically driven satellite-derived estimates and ground-level monitor data at a 1 × 1 km resolution.^26^ Annual NO_2_ concentration was estimated based on a national land-use regression using data from the Canadian National Air Pollution Surveillance (NAPS) monitoring network, coupled with data from satellites, road lengths within 10 km, areas of industrial land use within 2 km, and the mean summer rainfall.^27^ NO_2_ concentration was estimated at a 10 × 10 km resolution. The model explained 73% variation in the NO_2_ measurements from NAPS.^27^ Peak-season O_3_ was assessed with 21-km grid values and estimated based on the average of daily maximum O_3_ concentrations in the warm seasons (May 1 to October 31) and these estimates were used as the annual estimates for O_3_.^28^ The estimated annual pollutant concentrations were then spatiotemporally interpolated by scaling factors obtained from land-use regressions to calculate estimates of the weekly pollutant concentrations using information from monitors in the NAPS network and satellite data.^29^ The interpolation model showed large spatial and temporal coverage that is useful for national-scale longitudinal research on ambient air pollution.^29^

### Outcome Ascertainment

CP diagnosis was ascertained as (1) a single inpatient hospitalization diagnosis from CIHI-DAD (*International Statistical Classification of Diseases and Related Health Problems, Tenth Revision, Canada [ICD-10-CA])* code: G80), or 2 or more outpatient diagnoses at least 2 weeks apart on physician billing claims (modified *ICD-9* code: 343) using OHIP.^30^ Data for outcome ascertainment was available up to March 2020. Person-time of follow-up was calculated from birthdate to the first diagnosis of CP, loss to follow-up due to emigration from Ontario, death, or the end of follow-up (March 2020), whichever came first. The longest follow-up time was 18 years.

### Covariates

Demographic and delivery information were obtained from the databases as described previously and included maternal age at delivery, season of birth, calendar year of birth, and infant sex. Area-level socioeconomic status variables were obtained from ON-Marg and included indexes of community size, residential instability, material deprivation, dependency, and ethnic concentration.^31^ These marginalization indexes were first constructed from principal component analysis based on a broad range of indicators, and then encoded in categorical quintiles with a higher level of quintile suggesting the mother resided in a more marginalized area.^25^ Residential instability is the area-level concentration of people who experience high rates of family or housing instability. Material deprivation measures the ability for individuals and communities to access and attain basic material needs, including income, quality of housing, educational attainment, and family structure characteristics. Dependency is the area-level concentration of people who do not have income from employment. Ethnic concentration refers to area-level concentrations of people who are recent immigrants and/or people belonging to a visible minority group, which is defined by Statistics Canada as “persons, other than Aboriginal peoples, who are non-Caucasian in race or non-white in color.”^32^

### Statistical Analyses

Descriptive analyses were conducted for characteristics of the study population and distributions of ambient air pollutants during pregnancy. We calculated Pearson correlation coefficients for prenatal average ambient PM_2.5_, NO_2_, and O_3_ concentrations.

We performed multiple-pollutant Cox proportional hazards regression in combination with a distributed lag model (DLM) to estimate cumulative hazard ratios (CHR) as well as weekly hazard ratios (WHR) for CP per IQR increase in the cumulative or weekly ambient PM_2.5_, NO_2_, and O_3_ concentrations during pregnancy. Pollutant concentrations were standardized by their IQR to facilitate estimate interpretation. DLM allowed us to explore the weekly susceptibility of CP in relation to air pollutant exposure. Specifically, DLM incorporated average weekly concentrations of PM_2.5_, NO_2_, and O_3_ during gestational weeks 0 to 36 simultaneously as a cross-basis, which combined a linear dose-response function and a natural cubic spline time lag–response function with 3 degrees of freedom.^33,34^ The selection of degrees of freedom was based on model fitness by Akaike information criterion and visual inspections of the association patterns. The 3 pollutants were fitted as 3 separate cross-bases simultaneously in the model to control for confounding from each other. Models were adjusted for confounders identified a priori by a directed acyclic graph (DAG) (eFigure 2 in Supplement 1), and included maternal age at delivery, season of birth, fiscal year of birth, community size, quintile groups of residential instability, maternal deprivation, dependency, ethnic concentration, and infant sex.

Because sexually dimorphic differences have been identified and extensively studied in the CP literature, we further assessed heterogeneity by infant sex for the associations between prenatal ambient air pollutant exposure and CP risk. We conducted secondary analyses by infant sex and conducted likelihood ratio tests to obtain the *P* values for the interaction terms between sex and the cross-basis of each pollutant, which were regarded as the *P* values for heterogeneity by infant sex. Statistical significance was set at a 2-sided *P *value less than .05.

Because the majority of CP cases in the study population were diagnosed before age 3 years (80%) and more than 90% of cases were diagnosed before preschool (age 6 years), including cases diagnosed in later childhood or adolescence might dilute the observed association since it adds person-time at very low risk of CP diagnosis. Thus, as a sensitivity analysis, we restricted the Cox model (both main model and sex stratified) to CP cases diagnosed before age 6, adjusting for the previously mentioned covariates. Person-time of follow-up in this sensitivity analysis was calculated from birthdate to the first diagnosis of CP, age 6 years, loss to follow-up due to emigration from Ontario, death, or the end of follow-up (March 2020), whichever came first. Because ambient air pollution levels decreased over study years in Canada, we additionally examined heterogeneity by fiscal year at birth on the association between prenatal air pollutants and CP. Due to concerns over the potential collinearities among the 3 pollutants in the multiple-pollutant models, we fit single-pollutant models for the association between prenatal residential exposure of each pollutant and CP in sensitivity analyses. We further conducted multiple-pollutant models with higher degrees of freedom (4 and 5) to examine the robustness of the association shapes across pregnancy in the primary analyses. All data management steps and descriptive statistics were completed in SAS EG version 7.1 (SAS Institute). The dlnm package version 2.4.7 in RStudio version 3.3.0 (R Project for Statistical Computing) was used to perform multivariable regression modeling. Data were analyzed from January to December 2022.

## Results

### Descriptive Statistics

The present study included 1 587 935 mother-child pairs who reached term gestation, among whom 3170 children (0.2%) were diagnosed with CP. The median (IQR) age at time of CP diagnosis was 1 (0-3) year. Table 1 summarizes the characteristics of the study population for children with CP and children without CP. The overall mean (SD) maternal age at delivery was 30.1 (5.5) years, and mean (SD) gestational weeks at delivery were similar among children with CP (39.1 [1.2] weeks) and children without CP (39.2 [1.1] weeks) (Table 1). There was a higher percentage of male children among those with CP (1786 children [56.3%]) than among those without CP (809 959 children [51.1%]). Compared with children without CP, children with CP were slightly more likely to be born to mothers with a higher regional marginalization index and in smaller communities (Table 1).

**Table 1.** Characteristics of Full Term Births With and Without Cerebral Palsy (CP) Diagnosis Born in Ontario, Canada, 2002 to 2017

Characteristics	Participants, No. (%)		
	Births without CP (n = 1 584 765)	Births with CP (n = 3170)	Total (N = 1 587 935)
Individual-level characteristics			
Maternal age at delivery, mean (SD), y	30.11 (5.45)	29.76 (5.83)	30.11 (5.45)
Sex			
Male	809 959 (51.1)	1786 (56.3)	811 745 (51.1)
Female	774 806 (48.9)	1384 (43.7)	776 190 (48.9)
Gestational age, mean (SD), wk	39.22 (1.14)	39.05 (1.22)	39.22 (1.14)
Parity			
0	688 180 (43.4)	1485 (46.8)	689 665 (43.4)
1	585 717 (37.0)	1056 (33.3)	586 773 (37.0)
≥2	310 868 (19.6)	629 (19.8)	311 497 (19.6)
Season of birth			
Spring	400 585 (25.3)	788 (24.9)	401 373 (25.3)
Summer	420 049 (26.5)	828 (26.1)	420 877 (26.5)
Fall	398 563 (25.1)	791 (25.0)	399 354 (25.1)
Winter	365 568 (23.1)	763 (24.1)	366 331 (23.1)
Neighborhood-level characteristics			
Community size			
≥1 500 000	722 363 (45.6)	1309 (41.3)	723 672 (45.6)
500 000-1 499 999	205 229 (13.0)	358 (11.3)	205 587 (12.9)
100 000-499 999	380 945 (24.0)	869 (27.4)	381 814 (24.0)
10 000-99 999	121 391 (7.7)	311 (9.8)	121 702 (7.7)
<10 000	154 836 (9.8)	323 (10.2)	155 159 (9.8)
Instability quintile^a^			
1	330 798 (20.9)	572 (18.0)	331 370 (20.9)
2	294 645 (18.6)	616 (19.4)	295 261 (18.6)
3	283 971 (17.9)	578 (18.2)	284 549 (17.9)
4	312 851 (19.7)	642 (20.3)	313 493 (19.7)
5	362 500 (22.9)	762 (24.0)	363 262 (22.9)
Deprivation quintile^a^			
1	312 313 (19.7)	568 (17.9)	312 881 (19.7)
2	287 241 (18.1)	533 (16.8)	287 774 (18.1)
3	293 301 (18.5)	597 (18.8)	293 898 (18.5)
4	309 454 (19.5)	614 (19.4)	310 068 (19.5)
5	382 456 (24.1)	858 (27.1)	383 314 (24.1)
Dependency quintile^a^			
1	477 396 (30.1)	913 (28.8)	478 309 (30.1)
2	351 876 (22.2)	687 (21.7)	352 563 (22.2)
3	290 510 (18.3)	571 (18.0)	291 081 (18.3)
4	250 247 (15.8)	542 (17.1)	250 789 (15.8)
5	214 736 (13.6)	457 (14.4)	215 193 (13.6)
Ethnic concentration quintile^a^			
1	216 885 (13.7)	459 (14.5)	217 344 (13.7)
2	244 911 (15.5)	514 (16.2)	245 425 (15.5)
3	272 316 (17.2)	574 (18.1)	272 890 (17.2)
4	338 736 (21.4)	651 (20.5)	339 387 (21.4)
5	511 917 (32.3)	972 (30.7)	512 889 (32.3)
Levels during pregnancy			
PM_2.5_, median (IQR), ug/m^3^	8.3 (7.0-9.7)	8.6 (7.3-10.0)	8.3 (7.0-9.7)
NO_2_, median (IQR), ppb	13.0 (7.2-17.1)	13.4 (7.6-18.2)	13.0 (7.2-17.1)
O_3_, median (IQR), ppb	47.4 (44.0-51.1)	47.5 (43.6-51.9)	47.4 (44.0-51.1)

Abbreviations: NO_2,_ nitrogen dioxide; O_3_, ozone; PM_2.5_, fine particulate matter with a diameter 2.5 μm or smaller; ppb, parts per billion.

^a^ These quintiles have been created by sorting the marginalization data into 5 groups, ranked from 1 (least marginalized) to 5 (most marginalized).

The median (IQR) prenatal average concentrations of the 3 pollutants were 8.3 (7.0-9.7) ug/m^3^ for PM_2.5_, 13.0 (7.2-17.1) parts per billion (ppb) for NO_2_, and 47.4 (44.0-51.1) ppb for O_3_. Children with CP had slightly higher prenatal average ambient air pollutant levels than those without CP (Table 1). Prenatal average PM_2.5_ concentration was moderately correlated with NO_2_ (*r* = 0.57) but had a weaker correlation with O_3_ (*r* = 0.28), and the correlation between NO_2_ and O_3_ averaged across pregnancy was negative (*r* = −0.18).

### Associations Between Prenatal Ambient Air Pollution Concentrations and CP

A per IQR increase in the prenatal average PM_2.5_ concentration (2.7 ug/m^3^) across 37 gestational weeks was associated with an increased risk of CP (CHR, 1.12; 95% CI, 1.03-1.21) (Table 2). After stratifying the analyses by infant sex, we observed a statistically significant positive association between prenatal PM_2.5_ concentration and CP in male children (CHR, 1.14; 95% CI, 1.03-1.26) but not in female children (CHR, 1.08; 95% CI, 0.96-1.22), although the *P* value for heterogeneity was large (Table 2).

**Table 2.** Cumulative Hazard Ratios (HRs) and 95% CIs for Cerebral Palsy per IQR Increase in Pollutant Concentrations Among All Full Term Births and Male and Female Full Term Births (Results Obtained From Multipollutant Models)

Pollutant	Cumulative HR (95% CI)^a^			*P* value^b^
	All term births	Male births	Female births	
PM_2.5_ (per 2.7 ug/m^3^)	1.12 (1.03-1.21)	1.14 (1.03-1.26)	1.08 (0.96-1.22)	.85
NO_2_ (per 10 ppb)	0.93 (0.84-1.02)	0.95 (0.84-1.08)	0.90 (0.77-1.04)	.22
O_3_ (per 7 ppb)	0.97 (0.90-1.04)	0.93 (0.85-1.02)	1.03 (0.92-1.15)	.72

Abbreviations: HR, hazard ratio; NO_2,_ nitrogen dioxide; O_3_, ozone; PM_2.5_, fine particulate matter with a diameter 2.5 μm or smaller; ppb, parts per billion.

^a^ The model fitted the 3 pollutants simultaneously, and were adjusted for maternal age at delivery (continuous), season of birth (categorical), fiscal year of birth (categorical), community size (categorical), residential instability (categorical), quintile group of maternal deprivation (categorical), dependency (categorical), and ethnic concentration (categorical). Models fitted to all full term births were further adjusted for infant sex (binary).

^b^ *P* values for heterogeneity by infant sex were derived by likelihood ratio tests comparing the primary model with the model containing an interaction term between infant sex and the tested pollutant.

**Figure 1.**
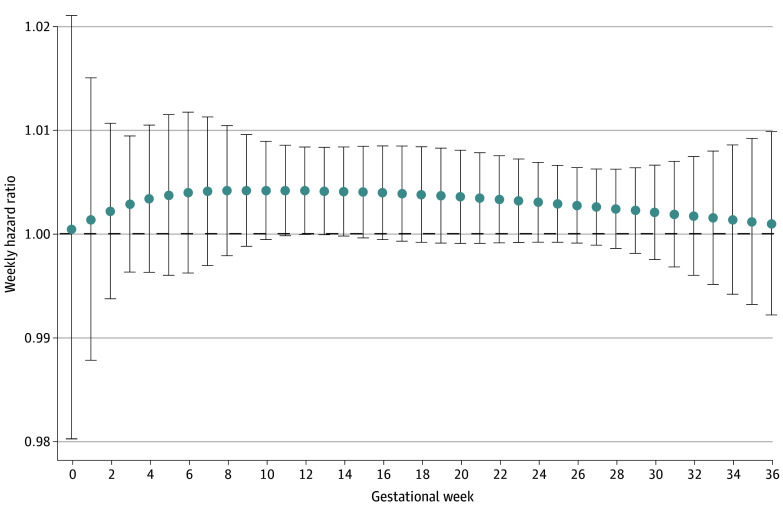
Weekly Hazard Ratio for Cerebral Palsy per IQR Increase in Prenatal Weekly Ambient Fine Particulate Matter With a Diameter 2.5 μm or Smaller (PM_2.5_) Concentration Among All Full Term Births The models fitted PM_2.5_, nitrogen dioxide, and ozone simultaneously and were adjusted for maternal age at delivery (continuous), season of birth (categorical), fiscal year of birth (categorical), community size (categorical), residential instability (categorical), quintile group of maternal deprivation (categorical), dependency (categorical), ethnic concentration (categorical), and infant sex (binary). Dots represent point estimates at each gestational week. Error bars indicate the respective 95% CIs.

**Figure 2.**
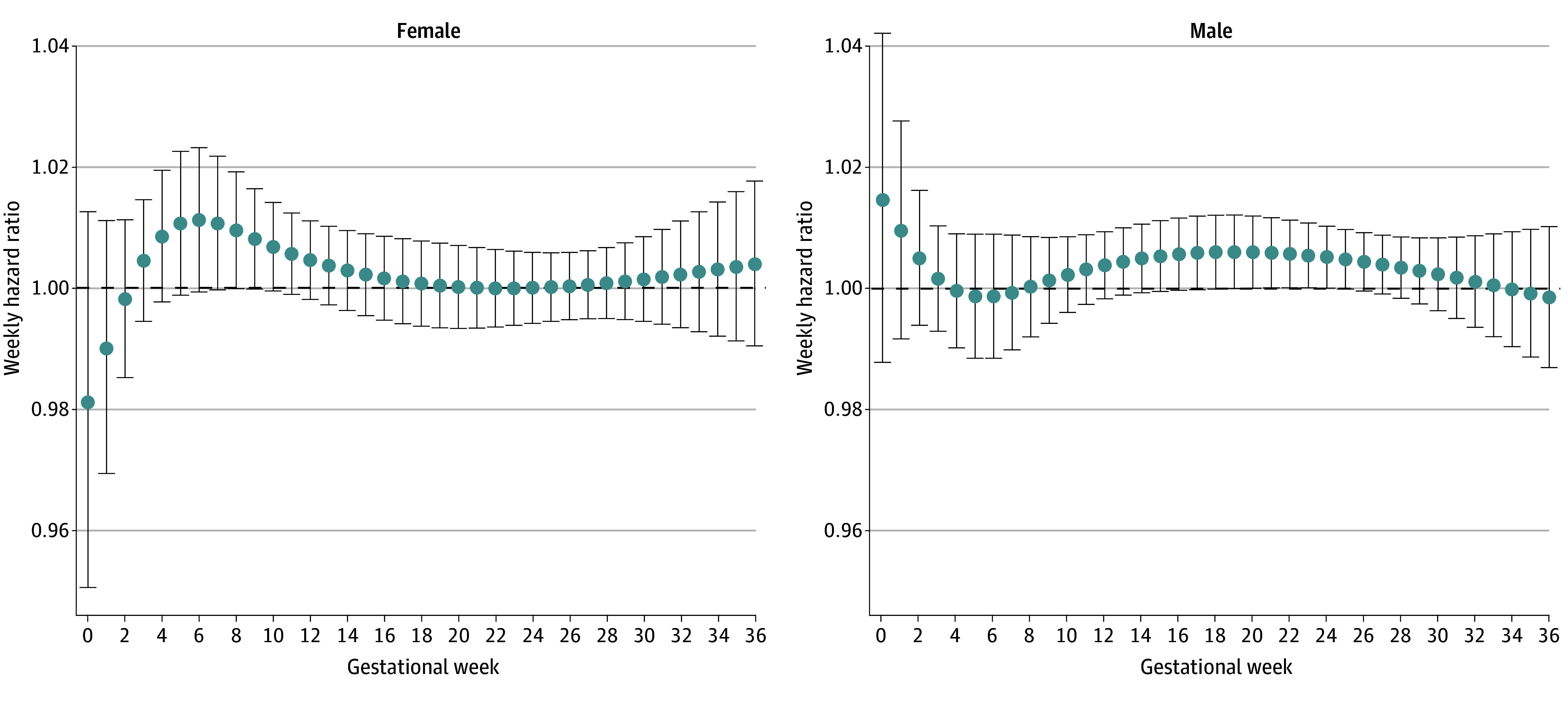
Weekly Hazard Ratio for Cerebral Palsy per IQR Increase in Prenatal Weekly Ambient Fine Particulate Matter With a Diameter 2.5 μm or Smaller (PM_2.5_) Concentration Among Male and Female Full Term Births The models fitted PM_2.5_, nitrogen dioxide, and ozone simultaneously and were adjusted for maternal age at delivery (continuous), season of birth (categorical), fiscal year of birth (categorical), community size (categorical), residential instability (categorical), quintile group of maternal deprivation (categorical), dependency (categorical), and ethnic concentration (categorical). Dots represent point estimates at each gestational week. Error bars indicate the respective 95% CIs.

Among all full term births, prenatal PM_2.5_ concentration was associated with higher hazards for CP across gestational weeks and no specific window of susceptibility was found (Figure 1). Among males, the point estimates for the WHR of PM_2.5_ and CP risk during gestational weeks 0 to 4 and 8 to 33 were higher than 1, with the highest WHR at gestational weeks 0 and 1 (WHR, 1.01) and 16 to 22 (WHR, 1.01), although the 95% CIs all crossed 1 (Figure 2). In female children, the WHR estimates of PM_2.5_ and CP risk were higher than 1 during gestational weeks 3 to 19, and estimates were highest during weeks 5 to 7 (WHR, 1.01), although all 95% CIs included 1 as similar to results found in male children (Figure 2). No overall association or windows of susceptibility were found for prenatal NO_2_ or O_3_ concentrations in relation to CP risks (Table 2; eFigure 3 in Supplement 1).

### Sensitivity Analyses

Restricting CP cases diagnosed before age 6 did not materially change the estimates (eTable 2 in Supplement 1). We did not find evidence of effect modification by birth year. Estimates from single-pollutant models were similar to those obtained from multiple-pollutant models (eTable 3 and eFigure 4 in Supplement 1). Results from multiple-pollutant models with higher degrees of freedom for the lag-response relationships showed similar overall patterns compared with the primary models (eFigure 5 in Supplement 1).

## Discussion

Average prenatal residential exposure to ambient PM_2.5_ was associated with an elevated risk of CP among full term births. The risk estimate in male infants was slightly higher compared with the estimate in female infants, although the difference did not reach statistical significance. No specific window of susceptibility was found among all full term births, but after stratification by infant sex, imprecise elevated CP risks were found for the weekly PM_2.5_ concentrations in the first and second trimesters among male infants and in the first trimester among female infants. We did not observe associations or sensitive windows for prenatal residential exposure to ambient NO_2_ or O_3_ in relation to the risk of CP.

Our results were consistent with the literature investigating the association between prenatal PM_2.5_ and other neurodevelopmental disorders such as autism spectrum disorder (ASD), attention-deficit/hyperactivity disorder (ADHD), and neurocognitive functions.^33,35,36^ Specifically, 1 study^35^ among a US Southern California cohort found ambient PM_2.5_ exposure in the first 2 trimesters was associated with increased risks of ASD from 2001 to 2014. Notably, prenatal PM_2.5_ concentration in this cohort is 1.7 times the concentration in our study population (14.2 vs 8.3 μg/m^3^). One study^36^ assessing prenatal NO_2_ exposure and gross motor and fine motor scores among 225 mother–full term birth pairs in Shanghai, China, did not find associations over the whole gestational period, which was consistent with the null result in our study. However, they suggested that weekly average NO_2_ concentrations had adverse associations with gross motor scores during gestational weeks 33 to 36, and with fine motor scores in weeks 26 to 36. The NO_2_ concentration in this Chinese study was twice that in our cohort (26 vs 13 ppb).

We found a slightly higher cumulative risk associated with prenatal PM_2.5_ exposure among male than female infants, although the *P* value for heterogeneity was large. This finding was consistent with the previous research showing male predominance in neurodevelopmental disorders, including ASD, ADHD, and CP.^37,38^ Additionally, we found no specific critical windows of exposure when analyzing all full term births together. However, after stratifying by sex, male infants had imprecisely elevated CP risk associated with ambient PM_2.5_ exposure in the early and middle pregnancy, while female infants were at higher CP risk in early pregnancy. The sex-specific difference in susceptible windows could be due to the sexually dimorphic susceptibility to specific CP subtypes which may have different vulnerable periods in utero but may also reflect the increased uncertainty in the weekly effect estimates.^38,39^ Nevertheless, the underlying mechanism of this male predominance and the sex difference in the windows of susceptibility warrant research to understand the underplay of genetic, hormonal, and environmental factors.

Some underlying mechanisms may help explain the findings. The fetal brain begins to develop during the third week of gestation,^23^ and cortisol neurogenesis takes place until the 24th week of gestation, which is characterized by proliferation, migration, and organization of neurons. Malformations at this stage such as cortical dysplasia are established causes of CP.^40^ Destructive injuries such as inflammation during brain development can also lead to CP.^40,41^ Myelination, which starts in the first trimester and is closely related to white matter disorders such as CP, is shown to be most susceptible to damage from particulate pollution.^10,20,42^ Animal model studies also suggest that particulate pollution is related to chronic neuroinflammation, which is also a potential cause of CP due to inflammation and oxidative stress via the placental pathway.^15,16,18,19^ An additional mode of mechanism could be through epigenetic changes, which is a recently hypothesized mechanism of CP,^43^ and prenatal air pollution has been shown to be related to differential DNA methylation.^44^ Mechanistic evidence supports the heterogeneity in inflammatory responses among male and female individuals, potentially explaining slightly higher CP risk related to PM_2.5_ exposure in male than female individuals. It was suggested that higher levels of proinflammatory cytokines (IL-1β, IL-6, and TNF-α) were observed in males, and were associated with less efficient and effective response when returning to homeostasis during acute inflammation.^45^ Animal studies also supported male predominance and found prenatal air pollution exposure could induce sexually dimorphic neuroinflammatory outcomes in adult offspring.^15,46^

### Strengths and Limitations

Our study has several strengths. First, using a Canadian provincial administrative database, this population-based cohort study captured sufficient sample sizes, person-time, and CP cases to achieve adequate statistical power, making this study the first and largest cohort study we know of on prenatal exposure to air pollution and risk of cerebral palsy. Moreover, since all the live births with valid exposure and outcome information in the province of Ontario were included in our study over almost 2 decades, our results could be generalizable to other populations of full term births with similar characteristics. Furthermore, by using the distributed lag models, we were able to estimate the weekly effect of prenatal exposure to air pollution, which enabled us to assess the granular windows of susceptibility, instead of a trimester-based manner, which may instead hinder some critical windows during fetal development. Additionally, we performed multiple-pollutant models which adjusted for the potential copollutant confounding.^47^ Since neither abnormal patterns nor estimates were found comparing results based on the multipollutant model vs single-pollutant model, we expected limited collinearity issues impacting the result. However, this study had some limitations. First, since the prenatal air pollution exposure was assigned by residence at delivery, there could be nondifferential measurement error in exposure assessment due to lack of consideration for residential movement during pregnancy, driving the observed associations toward the null and limiting our ability to detect vulnerable windows. The magnitude of measurement error is likely to be slight since mothers tend to move small distances during pregnancy.^48^ Second, we were unable to assess the subtypes of CP, which limited our ability to detect the associations and vulnerability windows of some specific subtypes of CP in relation to prenatal air pollution exposure. Third, although we adjusted for comprehensive variables of neighborhood socioeconomic status, residual and unmeasured confounding cannot be ruled out. Fourth, we limited our analyses to full term births, therefore, the findings may not be generalizable to those born preterm. Besides, there could be potential nondifferential outcome misclassification due to using administrative databases, potentially biasing the observed associations toward the null.^30,49^ Live birth bias may arise since we conditioned the analyses among live births, and prenatal air pollution was found to be related to pregnancy loss,^50^ which could potentially bias the association of interest downward.^51,52^ However, we would not expect the bias to be high given the relatively small effect of prenatal air pollution exposure on pregnancy loss.^50^ Additionally, the air pollution level in the Canadian population is one of the lowest in the world.^53,54,55^ Future studies with varying air pollutant levels are needed to validate our findings.

## Conclusions

In this large-scale population cohort, prenatal PM_2.5_ exposure was associated with an increased risk of CP. No specific window of susceptibility was found during pregnancy. The findings of this large cohort study could advance the identification of existing environmental risk factors for CP development and better inform interventions to mitigate the potential risk of CP during fetus development. Further studies are needed to validate the associations and explore potential modifiers.

## Notes

Supplement 1. **eTable 1.** Distribution of Gestational Age in Weeks at Birth Among Cerebral Palsy Cases of Full Term Births Born in Ontario 2002 to 2017 **eTable 2. **Cumulative Hazard Ratios and 95% CIs for Cerebral Palsy Diagnosed Before Age 6 per Interquartile Increase in Prenatal Ambient PM_2.5_, NO_2_, O_3_ Concentrations Among All Full Term Births and Male and Female Full Term Births **eTable 3.** Cumulative Hazard Ratios and 95% CIs for Cerebral Palsy per Interquartile Increase in Prenatal Ambient PM_2.5_, NO_2_, O_3_ Concentrations Among All Full Term Births, Results Obtained From Single-Pollutant Models **eFigure 1. **Flowchart of the Inclusion and Exclusion of the Study Population **eFigure 2. **Directed Acyclic Graph for the Associations Between Prenatal Air Pollution Exposure and Cerebral Palsy Risks **eFigure 3.** Weekly Hazard Ratio for Cerebral Palsy per IQR Increase in Prenatal Weekly Ambient NO_2_ and O_3_ Concentrations, Among All Full Term Births **eFigure 4.** Weekly Hazard Ratio for Cerebral Palsy per IQR Increase in Prenatal Weekly Ambient PM_2.5_, NO_2_, and O_3_ Concentrations and Cerebral Palsy Among All Full Term Births, Results Obtained From Single-Pollutant Models **eFigure 5**. Weekly Hazard Ratios for Cerebral Palsy per IQR Increase in Prenatal Weekly Ambient PM_2.5_, NO_2_, and O_3_ Concentrations and Cerebral Palsy Among All Full Term Births

Supplement 2.
Data Sharing Statement
