# Error in Funding/Support

**DOI:** 10.1001/jamanetworkopen.2024.39168

**Published:** 2024-09-18

In the Original Investigation titled “Prenatal Exposure to Ambient Air Pollution and Cerebral Palsy,”^1^ published July 9, 2024, there was an error in the Funding/Support listed. The article's Funding/Support section has been corrected to indicate that the study was supported by ICES, which is funded by an annual grant from the Ontario Ministry of Health and the Ministry of Long-Term Care. Additional language was added to the Disclaimer to clarify that analyses, conclusions, opinions, and statements included in the article are those of the authors and do not imply endorsement from organizations that provided the data.^1^
