# Supplementary material for: Prenatal Exposure to Ambient Air Pollution and Cerebral Palsy
Source: JAMA Netw Open. 2024 Jul 9;7(7):e2420717. doi: 10.1001/jamanetworkopen.2024.20717 (PMC11234239; doi:10.1001/jamanetworkopen.2024.20717)
Supplement: Supplement 1. — eTable 1. Distribution of Gestational Age in Weeks at Birth Among Cerebral Palsy Cases of Full Term Births Born in Ontario 2002 to 2017 eTable 2. Cumulative Hazard Ratios and 95% CIs for Cerebral Palsy Diagnosed Before Age 6 per Interquartile Increase in Prenatal Ambient PM2.5, NO2, O3 Concentrations Among All Full Term Births and Male and Female Full Term Births eTable 3. Cumulative Hazard Ratios and 95% CIs for Cerebral Palsy per Interquartile Increase in Prenatal Ambient PM2.5, NO2, O3 Concentrations Among All Full Term Births, Results Obtained From Single-Pollutant Models eFigure 1. Flowchart of the Inclusion and Exclusion of the Study Population eFigure 2. Directed Acyclic Graph for the Associations Between Prenatal Air Pollution Exposure and Cerebral Palsy Risks eFigure 3. Weekly Hazard Ratio for Cerebral Palsy per IQR Increase in Prenatal Weekly Ambient NO2 and O3 Concentrations, Among All Full Term Births eFigure 4. Weekly Hazard Ratio for Cerebral Palsy per IQR Increase in Prenatal Weekly Ambient PM2.5, NO2, and O3 Concentrations and Cerebral Palsy Among All Full Term Births, Results Obtained From Single-Pollutant Models eFigure 5. Weekly Hazard Ratios for Cerebral Palsy per IQR Increase in Prenatal Weekly Ambient PM2.5, NO2, and O3 Concentrations and Cerebral Palsy Among All Full Term Births [file jamanetwopen-e2420717-s001.pdf]

Supplemental Online Content

Zhang Y, Hu Y, Talarico R, et al. Prenatal exposure to ambient air pollution and cerebral palsy. *JAMA Netw Open*. 2024;7(7):e2420717. doi:10.1001/jamanetworkopen.2024.20717

eTable 1. Distribution of Gestational Age in Weeks at Birth Among Cerebral Palsy Cases of Full Term Births Born in Ontario 2002 to 2017
eTable 2. Cumulative Hazard Ratios and 95% CIs for Cerebral Palsy Diagnosed Before Age 6 per Interquartile Increase in Prenatal Ambient $PM_{2.5}$, NO_2, O_3 Concentrations Among All Full Term Births and Male and Female Term Births
eTable 3. Cumulative Hazard Ratios and 95% CIs for Cerebral Palsy per Interquartile Increase in Prenatal Ambient $PM_{2.5}$, NO_2, O_3 Concentrations Among All Full Term Births, Results Obtained From Single-Pollutant Models
eFigure 1. Flowchart of the Inclusion and Exclusion of the Study Population
eFigure 2. Directed Acyclic Graph for the Associations Between Prenatal Air Pollution Exposure and Cerebral Palsy Risks
eFigure 3. Weekly Hazard Ratio for Cerebral Palsy per IQR Increase in Prenatal Weekly Ambient NO_2 and O_3 Concentrations, Among All Full Term Births
eFigure 4. Weekly Hazard Ratio for Cerebral Palsy per IQR Increase in Prenatal Weekly Ambient $PM_{2.5}$, NO_2, and O_3 Concentrations and Cerebral Palsy Among All Full Term Births, Results Obtained From Single-Pollutant Models
eFigure 5. Weekly Hazard Ratios for Cerebral Palsy per IQR Increase in Prenatal Weekly Ambient $PM_{2.5}$, NO_2, and O_3 Concentrations and Cerebral Palsy Among All Full Term Births

This supplemental material has been provided by the authors to give readers additional information about their work.

© 2024 Zhang Y et al. *JAMA Network Open.*

eTable 1. Distribution of Gestational Age in Weeks at Birth Among Cerebral Palsy Cases of Full Term Births Born in Ontario 2002 to 2017.

Gestational week	Cerebral Palsy, n	Total Birth, N	Incidence, n/N
37	369	114327	0.32%
38	724	326635	0.22%
39	895	465989	0.19%
40	766	464015	0.17%
41	401	211439	0.19%
42	15	5387	0.28%
43	0	89	0.00%
44	0	35	0.00%
45	0	19	0.00%
Total	4644	1694106	0.20%

Note. We only included full term births, i.e., births born after 37 completed gestational weeks (⩾259 days), to our analyses.

© 2024 Zhang Y et al. *JAMA Network Open.*

eTable 2. Cumulative Hazard Ratios and 95% CIs for Cerebral Palsy **Diagnosed Before Age 6** per Interquartile Increase in Prenatal Ambient $PM_{2.5}$, NO_2, O_3 Concentrations Among All Full Term Births and Male and Female Full Term Births.

Pollutant	Cumulative Hazard Ratio (95% CI) [a]		
	All Full Term Births	Males	Females
$PM_{2.5}$ (per 2.7 ug/m3)	1.10 (1.01, 1.20)	1.13 (1.01, 1.26)	1.06 (0.93, 1.21)
NO_2 (per 10 ppb)	0.88 (0.72, 1.08)	0.84 (0.64, 1.10)	0.94 (0.70, 1.27)
O_3 (per 7 ppb)	0.95 (0.86, 1.04)	0.89 (0.79, 1.00)	1.03 (0.89, 1.20)

Abbrev. $PM_{2.5}$, fine particulate matter with a diameter ⩽ 2.5 μm; NO_2, nitrogen dioxide; O_3, ozone.
[a] The model fitted the three pollutants simultaneously. CP cases were diagnosed before age 6. Model was adjusted for maternal age at delivery (continuous), seasons of birth (categorical), fiscal years of birth (categorical), community size (categorical), residential instability (categorical), and quintile groups of maternal deprivation (categorical), dependency (categorical), and ethnic concentration (categorical). Models fitted to all full term births were further adjusted for infant sex (binary).

© 2024 Zhang Y et al. *JAMA Network Open.*

eTable 3. Cumulative Hazard Ratios and 95% CIs for Cerebral Palsy Per Interquartile Increase in Prenatal Ambient $PM_{2.5}$, NO_2, O_3 Concentrations Among All Full Term Births, Results Obtained From Single-Pollutant Models

Pollutant	Cumulative Hazard Ratio (95% CI) [a]
$PM_{2.5}$ (per 2.7ug/m^3)	1.08 (1.01, 1.14)
NO_2 (per 10 ppb)	0.98 (0.90, 1.07)
O_3 (per 7 ppb)	1.02 (0.97, 1.09)

Abbrev. $PM_{2.5}$, fine particulate matter with a diameter ≤ 2.5 μm; NO_2, nitrogen dioxide; O_3, ozone.

[a] All models were adjusted for maternal age at delivery (continuous), seasons of birth (categorical), fiscal years of birth (categorical), community size (categorical), residential instability (categorical), and quintile groups of maternal deprivation (categorical), dependency (categorical), ethnic concentration (categorical), and infant sex (binary).

© 2024 Zhang Y et al. *JAMA Network Open.*

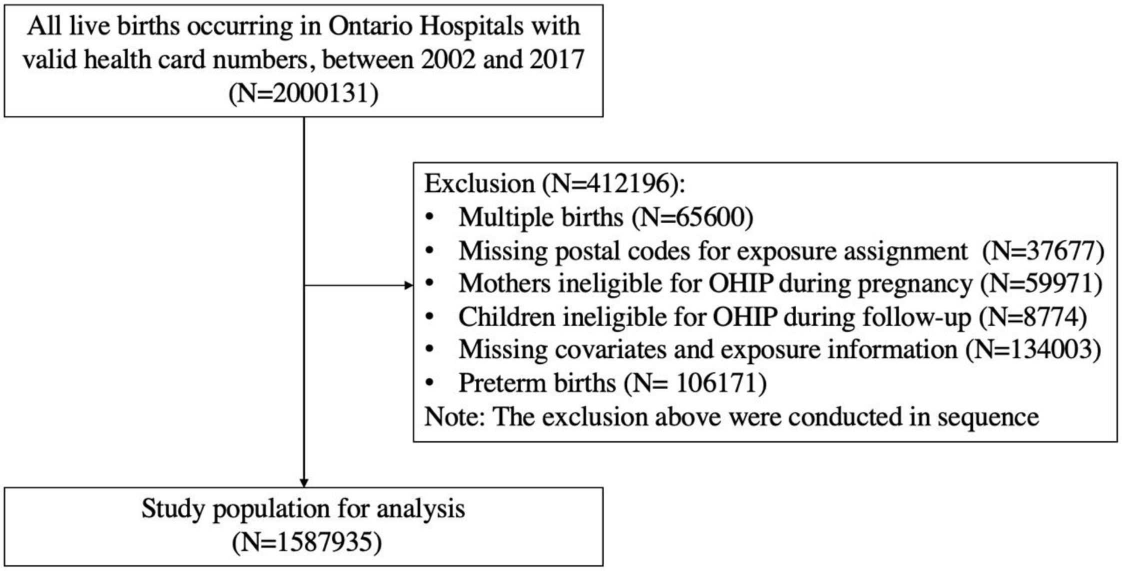

eFigure 1. Flowchart of the Inclusion and Exclusion of the Study Population.

© 2024 Zhang Y et al. *JAMA Network Open.*

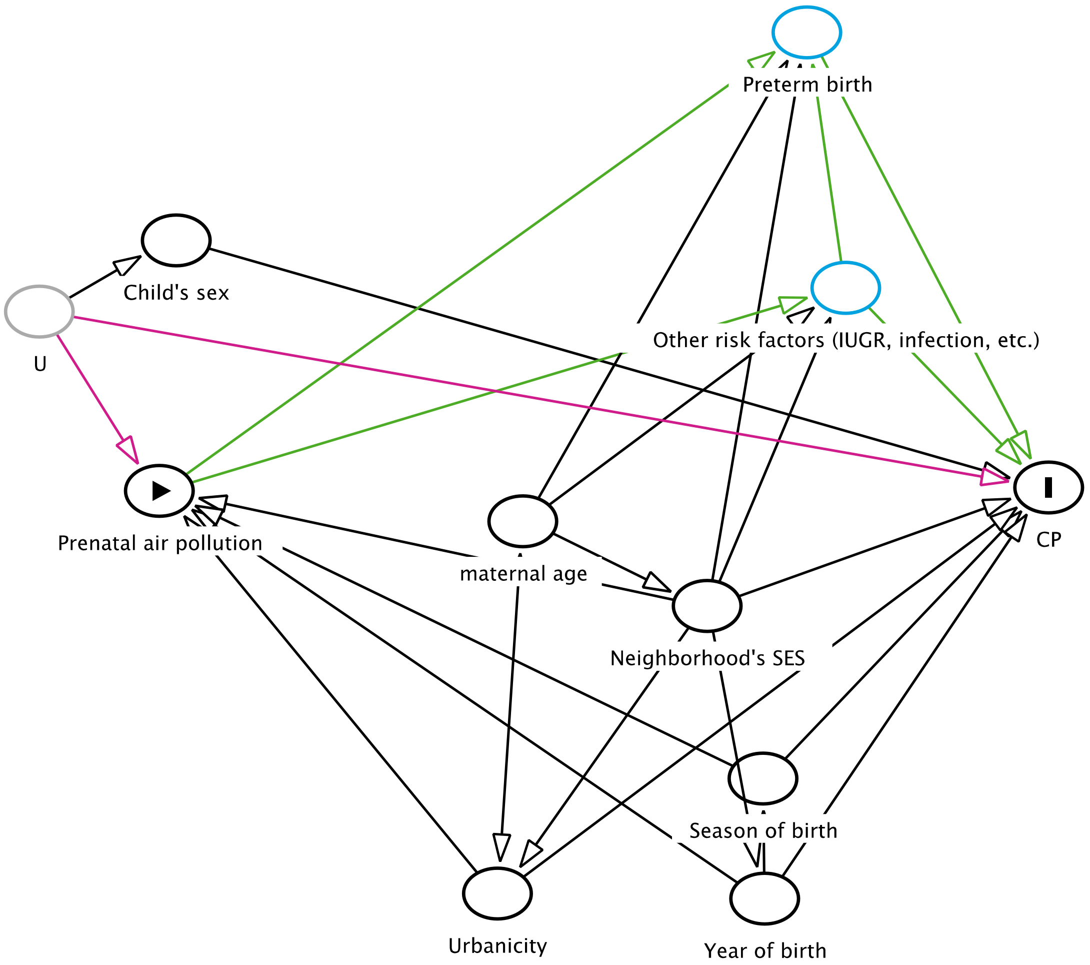

eFigure 2. Directed Acyclic Graph for the Associations Between Prenatal Air Pollution Exposure and Cerebral Palsy Risks.
Abbrev. SES, socio-economic status; IUGR, intrauterine growth restriction; CP, cerebral palsy.
Note. Black arrow lines represent controlled biasing paths. Red lines represent uncontrolled biasing path. Green lines represent causal paths.

© 2024 Zhang Y et al. *JAMA Network Open.*

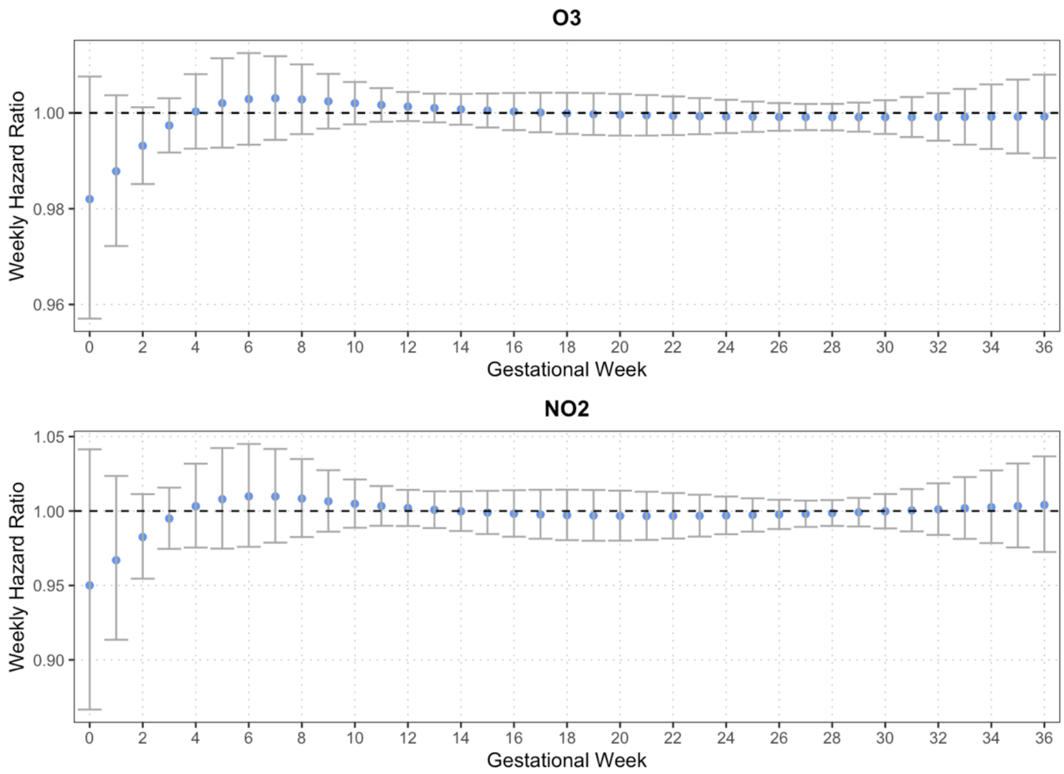

eFigure 3. Weekly Hazard Ratio for Cerebral Palsy per IQR Increase in Prenatal Weekly Ambient NO_2 and O_3 Concentrations, Among All Full Term Births.

Abbrev. NO_2, nitrogen dioxide; O_3, ozone.
Note: The models fitted the three pollutants simultaneously and were adjusted for maternal age at delivery (continuous), seasons of birth (categorical), fiscal years of birth (categorical), community size (categorical), residential instability (categorical), and quintile groups of maternal deprivation (categorical), dependency (categorical), ethnic concentration (categorical), and infant sex (binary).

© 2024 Zhang Y et al. *JAMA Network Open.*

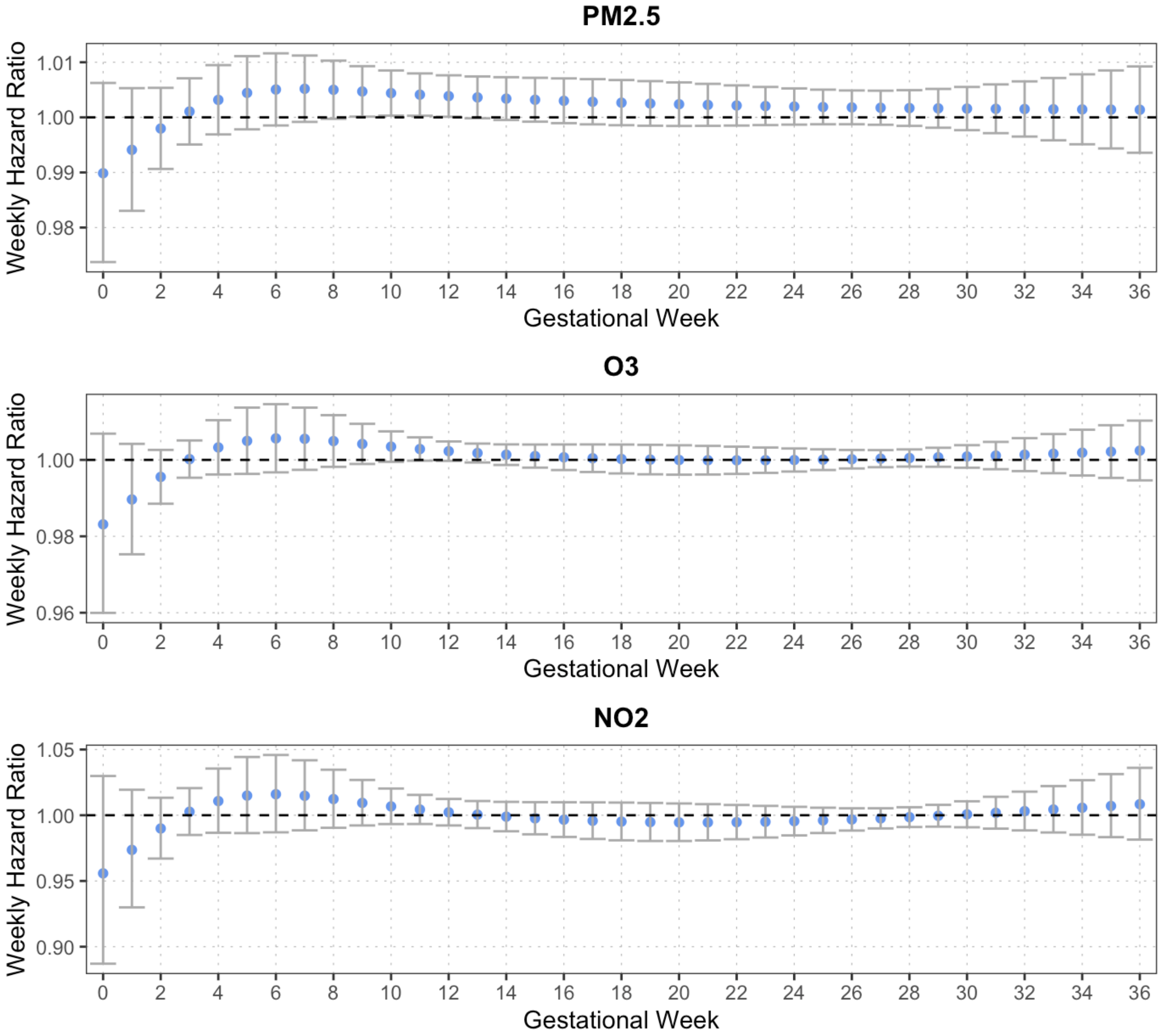

eFigure 4. Weekly Hazard Ratio for Cerebral Palsy per IQR Increase in Prenatal Weekly Ambient $PM_{2.5}$, NO_2, and O_3 Concentrations and Cerebral Palsy Among All Full Term Births, Results Obtained From Single-Pollutant Models.

Abbrev. $PM_{2.5}$, fine particulate matter with a diameter ≤ 2.5 μm; NO_2, nitrogen dioxide; O_3, ozone.
Note: All models were adjusted for maternal age at delivery (continuous), seasons of birth (categorical), fiscal years of birth (categorical), community size (categorical), residential instability (categorical), and quintile groups of maternal deprivation (categorical), dependency (categorical), ethnic concentration (categorical), and infant sex (binary).

© 2024 Zhang Y et al. *JAMA Network Open.*

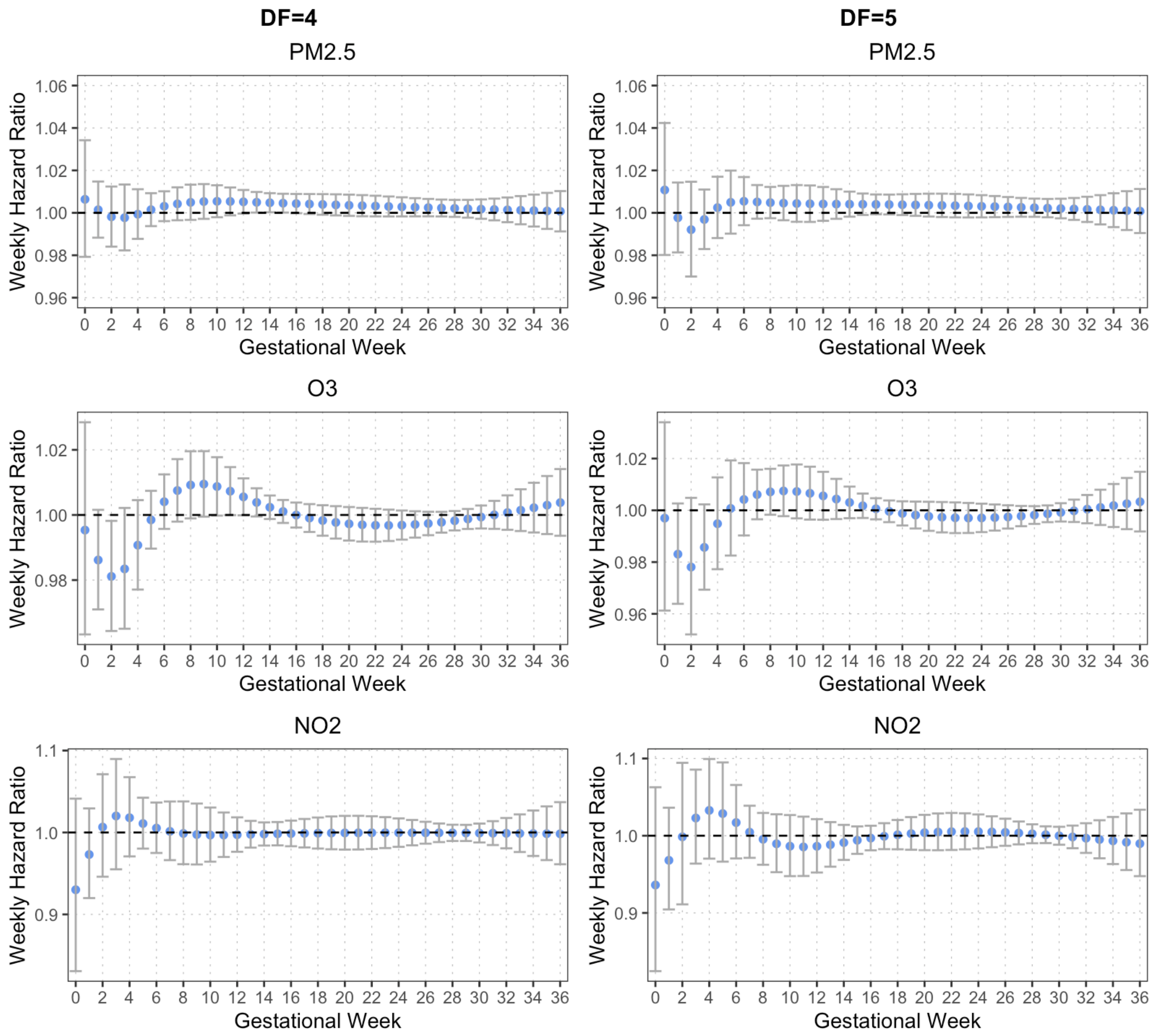

eFigure 5. Weekly Hazard Ratios for Cerebral Palsy per IQR Increase in Prenatal Weekly Ambient $PM_{2.5}$, NO_2, and O_3 Concentrations and Cerebral Palsy Among All Full Term Births, Results Obtained From Multiple-Pollutant Models With Degrees Of Freedom (DF) = 4 or 5 for the Lag-Response Relationship.

Abbrev. $PM_{2.5}$, fine particulate matter with a diameter ≤ 2.5 μm; NO_2, nitrogen dioxide; O_3, ozone; DF, degree of freedom.
Note: The models fitted the three pollutants simultaneously and were adjusted for maternal age at delivery (continuous), seasons of birth (categorical), fiscal years of birth (categorical), community size (categorical), residential instability (categorical), and quintile groups of maternal deprivation (categorical), dependency (categorical), ethnic concentration (categorical), and infant sex (binary).

© 2024 Zhang Y et al. *JAMA Network Open.*
