# Supplementary material for: Prenatal Exposure to Ambient Air Pollution and Cerebral Palsy
Source: JAMA Netw Open. 2024 Jul 9;7(7):e2420717. doi: 10.1001/jamanetworkopen.2024.20717 (PMC11234239; doi:10.1001/jamanetworkopen.2024.20717)
Supplement: Supplement 2. — Data Sharing Statement [file jamanetwopen-e2420717-s002.pdf]

Data Sharing Statement

Zhang. Prenatal Exposure to Ambient Air Pollution and Cerebral Palsy. *JAMA Netw Open*.
Published July 09, 2024. doi:10.1001/jamanetworkopen.2024.20717

Data
Data available: No

Additional Information
Explanation for why data not available: The data of this study is held securely at Institute of Clinical Evaluative Sciences. While legal data sharing agreements between Institute of Clinical Evaluative Sciences and data providers (eg, healthcare organizations and government) prohibit the institute from making the dataset publicly accessible, access may be granted to those who meet pre-specified criteria for confidential access, available at das@ices.on.ca">www.ices.on.ca(das@ices.on.ca). The full dataset creation plan and underlying analytic code are available from the authors upon request, understanding that the computer programs may rely on coding templates or macros unique to Institute of Clinical Evaluative Sciences and are therefore either inaccessible or may require modification.
